# The Wilderness Solo Experience: A Unique Practice of Silence and Solitude for Personal Growth

**DOI:** 10.3389/fpsyg.2020.547067

**Published:** 2020-09-10

**Authors:** Lia Naor, Ofra Mayseless

**Affiliations:** Department of Counseling and Human Development, University of Haifa, Haifa, Israel

**Keywords:** silence, solitude, nature based therapy, nature, personal growth, solo experience, wilderness, self reflection

## Abstract

Silence is now acknowledged by science as a significant construct of healthy human development and well-being, linked to humans’ neurobiology, psychology, physiology, and spirituality. This paper focuses on a particular form of silence experienced through the solo experience in the wilderness. The solo experience, involving varying periods of time spent in solitude and silence in the wilderness is a common method of intervention implemented among therapeutic and educational nature-based approaches. Numerous studies and personal accounts in the field underscore the solo experience as one of the most significant nature based interventions linked to various beneficial outcomes. These studies emphasize the significance of the wilderness, far from daily demands, and devoid of technological stimuli allowing the silence, time and space for self-reflection and contemplation on the sacredness and meaning of life. Although new to modern culture, solitude in nature is an ancient form of initiation used ceremonially by indigenous cultures worldwide. These practices challenge the individual who alone in the wilderness battles fear and loneliness only to discover inner strengths and true identity. The solo experience, viewed as enacting these ancient rituals in modern form may serve as an antidote to the loneliness, stress, and depression on the rise in the current era, which have been linked to our overly stimulated urban environments and lifestyles. This paper sheds light on how the wilderness solo is experienced and understood, specifically as contributing to therapeutic outcome and personal growth. The empirical and theoretical literature is reviewed pointing to the significance of solitude and silence as basic components of the wilderness solo. These are linked to profound personal outcomes including the discovery of new and expansive ways of knowing the self and the world, specifically as interconnected in the larger web of life, enhancing a sense of personal belonging and purpose.

Nobody can counsel and help you, nobody. There is only one single way.

Go into yourself.

—Rainer Maria Rilke

## Introduction

Silence is acknowledged to be a human quality or value that is important in a variety of significant areas of psychological development (Denham-Vaughan and Edmond, 2010; Kemerer, 2016; Vaughan and Klimo, 2016). Scholars and researchers underscore the many opportunities embedded in silence for human beings to explore the contents of consciousness and gain insight, free from environmental and social constraints (Lehmann, 2016; Lehmann et al., 2019). However, in modern life, immersed in noise, commotion, and stimuli, it may be hard to experience silence and further to acknowledge its benefits, especially when many individuals associate silence with unwanted feelings like loneliness and anxiety (Maxted, 2005).

This paper focuses on the facilitated *wilderness solo experience*, defined broadly by Knapp and Smith (2005) as a retreat from daily life, where the individual spends a prescribed time alone and in silence, in the wilderness, to contemplate the meaning and purpose of life, in a way that is difficult to do in everyday settings. The wilderness solo experience is commonly offered by counselors, psychotherapists, coaches, and educators, as well as by outdoor adventure leaders, and has gained empirical and professional credibility as one of the most influential components of outdoor education, contributing to participants’ learning and growth (Bobilya et al., 2005; Daniel, 2005; Kalisch et al., 2011). The solo experience offered by professional practitioners as part of a therapeutic program, can be considered a unique nature-based intervention (NBI) providing individuals with a secure and professional framework to experience silence and solitude in the context of personal growth (Shanahan et al., 2019).

Silence has been underscored as a central characteristic of the solo experience in the wilderness, but the characteristics, effect, and personal experience of silence within this context have yet to be explored (Knapp and Smith, 2005), Literature in the field of health psychology has focused on silence in the context of therapeutic intervention, mostly as the absence of verbal communication, in individual psychotherapy, psychoanalysis, and group therapy (Elson, 2001; Levitt, 2001; Ladany et al., 2004). Hence, our understanding of the effect that the practice of silence has on the nature and quality of inner experience and human behavior in the field of psychology is limited (Valle, 2019). Despite growing evidence that attests to its merit (see, for example, Kalisch et al., 2011, extensive review), the wilderness solo experience, perceived as a distinct therapeutic intervention, integrating silence and solitude, has yet to gain attention within the broad field of psychology (Nicholls, 2008). This paper seeks to gain further understanding regarding the therapeutic value of silence by focusing on the wilderness solo experience as a unique growth-oriented intervention. A brief review of the study of silence in the psychological and therapeutic context is presented, followed by a description of the method of inquiry of this study, which builds upon a review of theoretical and empirical literature in order to discuss the basic components of the wilderness solo experience—silence, solitude, and the wilderness—in the context of personal growth. We conclude with a statement of the significance of the study, as well as its limitations.

## Silence

*Silence is not the absence of something but the presence of everything.* —Gordon Hempton

Although silence has commonly been defined as the absence of sound or communication (Valle, 2019), current discourse has examined silence more as a path allowing the presence and development of human qualities. In his work on the nature of silence, Picard (2002, p. 15) described silence as “an autonomous phenomenon [which is] not simply what happens when we stop talking [but] an independent whole, subsisting in and through itself.” Therefore, it is through the absence of external noise that a profound process of gaining knowledge may be triggered (Picard, 2002).

The significance of silence to human experience has propelled inquiry in the realm of psychology and mental health, revealing both positive and negative aspects of silence. Lehmann (2016) utilizes the typology of silence proposed by Bruneau and Ishii (1988), denoting that silence-related phenomena fall into one of three categories: (a) silence—a solitary and mystical experience, often described as the temporal dissolution of the perception of time and space; (b) silences—social aspects of integration, connection, and communication; or (c) silencing—as a means of restricting someone else’s expression by exhorting power. Although these three notions are interconnected, this paper focuses on the first category—the value of silence as a human quality linked to self-discovery and inner knowledge, defined by Lehmann et al. (2019) as being aware of, perceiving, thinking of, and analyzing the contents that show up in consciousness.

Through this lens, silence has been found to be a significant and active factor in the therapeutic process. Elson (2001, p. 351) reviewed the various types of silence occurring in it, emphasizing the opportunity for change and growth embedded in periods of silence. She described silence in the therapeutic dialect as a powerful tool that, when worked with effectively, may allow immersion in the creative experience of restoring, renewing, or initiating strengths and capacities inherent in a cohesive self. She concluded that “silence itself is a fertile mode in which the self is enriched and strengthened, the source of that quiet growth in which distortions of the self can be reflected upon and then transformed.”

Dénommé -Welch and Rowsell (2017, p. 23), in their review of the epistemologies of silence, suggest that silence may support personal growth and development. The authors define silence as “providing individuals with a fluidity and mobility to shift moods and environments and as such is transitional and it invites change.” Marcandonatou’s (1998, p. 318) study, which investigated the experience of “being voluntarily silent for a period of four or more days” by implementing a variation of classic existential–phenomenological methodology, revealed nine comprehensive themes that represent transpersonal dimensions of existence portraying the research participants’ shift “from their personal ego-self perspective toward aspects of experience that can be named transegoic.” In light of her findings, Marcandonatou concludes that silence may be linked to personal transformation, union, transcendence, mystical states, and love.

The opportunity embedded in silence for positive change and human development was the focus of Valle’s (2019) metareview of the behavioral and experiential approaches to silence in psychology and the effects that intentionally practicing silence have on deepening one’s experience. Based on his extensive review, Valle (2019) proposed a classificatory system of ten forms of silence, ranging from the most external worldly manifestations to the subtlest, most inwardly attuned discernments (environmental, sensory, psychic sensory, emotional, verbal, mental, pre-reflective, intuitive, ontological, and transcendent), illuminating the human potential to experience vast dimensions of human existence through various forms of silence.

These conceptualizations emphasize the psychological value of experiencing silence, which is especially relevant in the modern era characterized by intense lifestyles that leave us with very little space, if any at all, to experience internal and external quiet (Bobilya, 2005). Burns (2005) stated that it is the diversion from silence that has distanced us from gaining a deeper knowledge of self. Indeed professionals from various areas of interest posit a fundamental psychobiological mismatch between humans and their largely non-natural, technological environments and lifestyles (Byrnit, 2006). More than five decades ago, Jung (1951) stressed that as a result of the modern lifestyle, devoid of quiet introspection and reflection, “our psyche is profoundly disturbed,” and the boundless activities characterizing people’s daily lifestyles have led to extraverted and overstrained individuals (in Sabini, 2008, p. 255).

The opportunity to slow down and reflect in silence may be exactly what is needed to find internal peace in the busyness of our lives (Bobilya, 2005). Czech philosopher Kohak (1984) referred to silence as a gift that we need to reclaim if we are to become fully realized humans. The ancient tradition of wilderness solitude, revived in the current age as the wilderness solo experience, may provide individuals with a path to practice the gift of silence.

### The Wilderness Solo Experience as a Path to Personal Growth: From Ancient Practice to Modern Times

The wilderness solo experience derives its roots from ancient traditions in which people sought healing, rejuvenation, self-knowledge, and spiritual insight, gained through journeys and retreats into the wilderness, often alone (Storr, 1988; Gibbens, 1991; Fredrickson and Anderson, 1999). Moses, Jesus, Buddha, and Gandhi are examples of great leaders who regularly spent time alone in the wilderness that was associated with their personal transformation and enlightenment (McDonald and Schreyer, 1991).

Throughout history and across cultures, removing oneself from the demands of daily life in order to contemplate life in the silence and solitude of the wilderness has been practiced in various forms. The *vision quest* is a common form of this practice. Nineteenth-century anthropologists and cultural historians first used the term *vision quest* to describe a ceremonial initiation practiced among ancient traditions, by which the individual journeyed into the wilderness for an unlimited time alone to find their individual strengths, calling, vision, and purpose in life (Gennep, 1960). Traditionally, the quest began by purifying the body, mind, and spirit and concluded with the community welcoming the individual back, commemorating the triumph by adorning him or her with a new title or role in the community (Smith, 2005a). The vision quest marked what Arnold van Gennep (1960) defined as a *rite of passage*, a universal phenomenon of human development enacted through initiation rituals involving the common sequence of separation, transition, and incorporation. In many cases, a quest into the wilderness alone was taken to mark the transition from one life stage to the next, by rising to a challenge of body, mind, soul, and spirit (Norris, 2011).

This tradition of retreat into the wilderness to gain insight, guidance, and inspiration has led to the development of the modern-day, facilitated *wilderness solo experience* as a common intervention in the field of wilderness therapy and outdoor education (Norris, 2011). In their book *Exploring the Power of Solo, Silence, and Solitude*, Knapp and Smith (2005) define the wilderness solo experience as a retreat alone into the wilderness for a prescribed time (typically several days), sometimes fasting, allowing the individual to reflect on and better understand their place, purpose, and direction in life. The wilderness solo experience is usually a facilitated group process, comprised of a significant period for preparation, followed by time alone (from several hours to several days) in a specific designated place in the wilderness, concluding with integration of the experience in the context of the group. Variations of the solo experience may include physical activities, such as hiking or canoeing, which may or may not be part of a facilitated program, and solo experiences that are implemented alone rather than in a group.

Wilderness solo experiences are widespread in outdoor and adventure programs for youth worldwide, such as Outward Bound and the National Outdoor Leadership School, and are perceived not as a survival exercise but rather as a profound growth and learning experience (Smith, 2005a). Solo experiences in the wilderness are also prevalent in various nature-based programs focusing on personal growth for adults from the general public. One example is The School of Lost Borders, founded in 1984 by Steven Foster and Meredith Little, which has facilitated thousands of people through wilderness solo experiences. The wilderness solos they offer adhere to the three basic phases of the ancient rite of passage, but while in indigenous cultures the individual would go through these phases alone and each phase could go on for an unlimited amount of time, in contemporary wilderness solos these stages are limited and facilitated by experienced guides. These programs typically involve several days of preparation during which the participants spend time together participating in ceremonial practices designed to help them prepare for the journey as much as possible. Norris (2011), who explored rites of passage models in adventure therapy, delineates the stages: The first stage involves the preparation for the solo, which includes talks and exchanges in the group about safety issues, physical challenges, aspects of the flora and fauna in the area, and emergency procedures. A considerable part of the preparation is also dedicated to potential psychological challenges, personal fears, and clarification of the participant’s personal intention for participating in the solo. This procedure rests on the assumption that the more aware the participants are of the feelings that can arise as part of the experience and of their personal intention the more they can learn from the experience (Knapp and Smith, 2005). The second stage is the initiation, typically involving three days of solitude and fasting in the wilderness. What happens during this time is highly individualized—some participants relay experiences of connecting and conversing with nature, the elements, and animals; others experience boredom, hunger, and loneliness; some experience deep love and joy; and for many this is a time to evaluate their relationships and lives (Norris, 2011). In this stage, participants are encouraged to contemplate their life’s purpose (e.g., vision) and to consciously, and preferably ceremonially, mark what is ending in their life while apprenticing to a new beginning. Incorporation is the last stage, beginning with the individual’s return to the group and the everyday world and the processing of the experience. Usually, participants are welcomed with a generous meal to break the fast and after a period of rest the group gathers in circle to start sharing their experiences. Here, the program guides take on the role of the elders in indigenous traditions as they listen to the stories and mirror back elements of significance and meaning of the soloists’ stories (Smith, 2005b). The incorporation phase focuses on how the solo experience and insights gained can be applied to the participant’s life toward positive change and profound growth.

Variations of solo time spent in silence in the wilderness are prevalent in nature-based programming worldwide and are perceived as a key factor in gaining educational and therapeutic outcomes (Knapp and Smith, 2005). A growing body of literature points to the solo experience in the wilderness as a unique experience linked to profound therapeutic outcomes (Daniel, 2005; Knapp and Smith, 2005; Coburn, 2006; Nicholls, 2008; Kalisch et al., 2011). This paper reviews the literature in the field, highlighting the facets of the wilderness solo experience that are relevant to understanding its value in the context of personal growth.

## Method

The solo experience may be viewed from various perspectives (e.g., psychological, social, or cultural). To gain a better understanding of the therapeutic value embedded in the solo experience, literature from the fields of leisure studies, outdoor education, and nature-based therapies was reviewed, including books, personal accounts, and research studies that focused on solitude and silence experienced in the wilderness. This review was not limited by the year of publication of materials or the framework of intervention involved. Through this expanded lens, various forms of the solo experience were assessed, including what authors and researchers referred to as *solo time* or *solo experience* denoting periods of time spent in solitude and silence in the wilderness that were part of a designed program for youth as in Outward Bound. We also reviewed studies and accounts of time spent alone in silence as part of nature-based workshops for adults defined by the terms *vision quest* or *rite of passage*. In light of the limited empirical data on the topic, we expanded our initial focus on the facilitated solo experience to include various studies that focused on the experience of silence and solitude in the wilderness that were or were not part of a facilitated program. These included accounts of wilderness excursions, hiking, or canoeing alone for a period of time that provided us with important understandings regarding the value of experiencing silence and solitude in the wilderness. Additional studies focusing on solitude from the psychological literature were reviewed when current literature was not sufficient.

### Empirical Review

Qualitative methods are the prominent methodology in the study of the wilderness solo experience. They provide us with an understanding regarding the subjective, lived experiences of participants in the wilderness solo (Nicholls, 2008). Various qualitative studies have been conducted with adults who experienced different forms of wilderness solitude, including wilderness immersion (Hammitt and Brown, 1984), canoeing (Swatton and Potter, 1998), hiking (Fredrickson and Anderson, 1999), solitude (Coburn, 2006), and modern vision quest ceremonies (Wilson, 2011). These studies stress the significance of the natural environment, devoid of daily commotion, human interference, and mental and emotional demands, allowing a specific form of silence contributive to self-reflection and profound insight (Knapp and Smith, 2005).

This unique form of self-reflection, experienced by spending time alone in the wilderness, was the focus of Hammitt and Brown’s (1984) inquiry into the cognitive dimensions of wilderness solitude. The researchers developed a wilderness privacy scale to identify and measure the various dimensions and functions of privacy among wilderness users. The 28-item questionnaire was submitted to 109 university students in outdoor recreation classes. Their findings indicate emotional release and resting of the mind from anxiety and mental fatigue, as linked to cognitive change attained through privacy and silence in the wilderness. The researchers concluded that wilderness privacy allows for the integration of one’s thoughts and experiences, which is key for releasing stress and efficient functioning (Hammitt and Brown, 1984). The wilderness privacy scale was examined by Hammitt and Madden (1989) among 184 overnight backpackers in Great Smoky Mountains National Park. Factor analysis of twenty items produced five main factors, ranging from most important to least: tranquility and the natural environment, individual cognitive freedom, social cognitive freedom, intimacy, and individualism. The natural environment, free of artificial noise and intrusions, was found to provide a sense of tranquility and peacefulness. The researchers concluded that wilderness privacy is a much more complex concept than being alone, which they described as a form of privacy in a specific mental setting where individuals experience an acceptable and preferred degree of control and choice over the type of information they must process (Hammitt and Madden, 1989). The findings of these studies were supported eighteen years later in Hammitt et al. (2001) study, conducted as a replication and comparison of cognitive perceptions found in Hammitt and Brown’s (1984) original study. The cognitive dimensions of wilderness solitude were assessed by administering the same questionnaire and data analysis to the same number (n = 109) of university students who spent time alone in nature backpacking. Cognitive states of solitude were illuminated as involving mental renewal attributed to rest from anxiety and mental fatigue. The participants attributed this renewal to the independence, individuality, and self-evaluation described as functional attributes of the wilderness, devoid of manipulation and domination from others.

Swatton and Potter’s (1998, p. 15) qualitative study examined the link between wilderness solitude and personal growth. Four expert North American canoeists, ages 45 to 68, who had completed four or more solo canoe expeditions of two weeks or longer, were interviewed to understand their solitary wilderness experiences. Their findings highlighted the significance of the solo experience as involving three main components: (a) being alone in silence, which allowed the tranquility, peace, and time necessary for self-reflection; (b) the physical, mental, and emotional demands of the canoeing trip, which allowed the paddlers to become more aware of themselves in relation to the natural surroundings and propelled a sense of self-actualization; and (c) the natural environment, which provided unscheduled time devoid of disturbances or a sense of judgment by other people, allowing them to observe and explore the self with greater freedom of expression. The researchers concluded that the wilderness solo experience is a “powerful environment for individuals to become aware of their own potentials, capabilities, and talents and that solitude encourages individuals to explore, discover and actualize their potentials.” These studies did not focus on silence specifically, but they did emphasize how the natural environment provided a unique form of social, mental, and environmental quiet or silence, conducive to self-reflection and mental clarity linked to personal growth.

The only study focusing on the sensed quality of silence and solitude in the wilderness solo is Nicholls (2008) qualitative study. Nicholas examined the internal and subjective “sense” of silence and solitude, defined as “quiet time.” Eighteen students, males and females, who participated in the solo experience as part of a wildernesses therapy program, were interviewed, and data was collected in the form of journals and field notes. The wilderness solo emerged as involving a subjective sense of solitude, contrary to common conceptions that define solitude as an objective and external condition (Larson, 1990). The findings of this study revealed four co-occurring subjective conditions by which the sensed experience is defined: (a) a sense of being alone, (b) a positive mind frame, (c) a personal time perspective, and (d) focused attention. The positive experience and effect of the solo experience was attributed to an individual experience of solitude and silence, shared by a small social group. This led to the definition of the solo experience as *being alone together*, a term initially coined by Hammitt (1982). In the social and environmental context, the time spent in ruminative reflection and/or simply focusing on nature had a positive impact on participants’ understanding of themselves and on their capacity to understand some of their unresolved and significant domestic concerns.

In Foster and Borrie’s (2011, p. 7) study, the significance of the distinct *quiet time* spent alone in the wilderness lay in the connections cultivated in that space, between the participants and the natural environment. Interviews were conducted with thirty-two overnight backcountry campers, ranging from nineteen to sixty-seven years old, traveling alone by canoe. Analyses of the interviews revealed distinct ways of engagement with nature; the participants immersed themselves in simple ways of being and escaped technological information that was often said to have taken over their everyday lives. The natural environment, described by the participants as free from intentional human control, provoked new ways of relating to themselves, other people, powers greater than themselves, and the wild landscape. The majority of participants emphasized the mental calm and self-reflective thinking brought on by the wilderness. The researchers concluded that in nature, far from everyday routine, social constraints, technology, and daily duties, one has “the time and space to re-connect with others and with the greater creation.” In these conditions, the participants’ relationship with an array of spiritual themes was often kindled and/or sustained.

Experiencing a significant relationship and connection with oneself and the environment through solitude in the wilderness has been shown to elicit significant outcomes and, in many cases, even personal transformation. Coburn (2006) explored the nature of psychospiritual transformation experienced among twelve women hiking alone over 2,000 miles on the Appalachian Trail. Extensive review and analysis of the data, including personal accounts written by the participants, spoken stories about their experience of solitude in the wilderness, and created or chosen visual images of transformation shed light on the transformative aspects of the experience. The constructs of personal transformation that emerged included experiencing a sense of wonder, feeling competent, trusting, a sense of being fully present in the moment, becoming more authentic, and desiring to be of service. The participants attributed these constructs to the time spent alone and in silence, allowing them to experience acceptance and interconnectedness in the vast, timeless, and ever-changing natural environment. In these conditions, the participants developed a new and intimate connection with self, others, and nature that led to what they described as the dissolving of their former identity and the development of a more connected and authentic one.

Similar findings appear in Wood’s (2010) research on self-evaluated psychospiritual transformation among twelve adults who participated in modern-day wilderness solos. The transformative constructs of the participants’ experience included: (a) experiencing significant connections with nature through interaction or relationship, described as a sense of being a part of nature and being guided by nature instilling a sense of purpose, (b) a shift in awareness, described as a shift from a state of questioning to a state of understanding, and (c) self-acceptance, involving the recognition of previously unrecognized parts of the self. The personal transformation was described as feeling more authentic or whole, discovering a purpose in life, and the ability to embrace life in its fullness. Such transformation involved the integration of newly discovered aspects of themselves revealed as a result of connecting with nature during the wilderness solo. This realization was attained through experiencing nature as guiding and enabling, experiencing non-separateness with the wilderness, experiencing a range of significant feelings through encounters with nature, experiencing nature as sacred, viewing inner processes as part of nature, experiencing flow states of consciousness in nature, and being passionate about maintaining a conscious relationship with nature. Being oneself in a more authentic way, feeling more serene and peaceful, and securing a purpose in life comprised the psychospiritual transformations the participants experienced. Importantly, this study reveals that modern-day wilderness solos provide the opportunity for the kinds of experiences that invite such psychospiritual change.

Unfortunately, most of these studies have been conducted with small population groups and thus have been limited in scope. Therefore, Kalisch et al.’s (2011) study of the solo experience as conducted in Outward Bound and undergraduate wilderness programs in the United States is seminal in providing us with further understandings. For almost two decades, the researchers examined participants’ experiences of the solo in wilderness programs using multiple methods, including written surveys, focus group interviews, one-on-one interviews, and field notes; in some cases participants were asked to reflect on how the solo had affected them a year after the program. The 335 first-year college students who participated in solos of various lengths (twenty-four to sixty hours) as part of eighteen-day wilderness programs described the solo as the most significant aspect of the wilderness experience. The researchers assessed the components attributed to personal growth attained through the solo experience relating to three components. The first component was the participants’ receptivity. Most students documented feeling excited upon entering the solo, and most of the students chose the word *solitude* when asked to identify the most enjoyable characteristic. Taken together, these factors suggest receptivity upon entering and during the experience, which often contributes to personal growth. The second component was described as optimum stress related in the context of personal growth to overcoming challenge. This suggests an explanation for the number of participants who responded positively to solitude, fasting, inactivity, or unstructured time. Although these experiences were often described as the most difficult, they were also valued or most enjoyed by some participants. The stress did not diminish the quality of their solo; rather, it was facilitative of their personal growth. The third component involved change and attunement. The data showed that as the solo progressed, the participants’ attunement to their own lives, to relationships with others, and to the natural environment was also enhanced. The results indicate that although participants valued the time alone, they also found these to be the most difficult aspects of the solo. Solitude was simultaneously the most enjoyable and the second most difficult aspect of the solo, followed by boredom and preceded by fasting. Within this context the wilderness solo was an opportunity for the participants to become aware of spiritual and/or religious dimensions of life and to clarify, evaluate, and redirect themselves by setting goals for the future. By reflecting on themselves in relation to the wilderness, others, and, in some cases, God, participants became more attuned to the important matters in their lives and in the world of which they were a part.

The findings of these studies highlight the significance of self-reflection and contemplation experienced in the wider context of the wilderness. Daniel’s (2005) retrospective study supports this, finding the wilderness solo to be a significant life experience, related to the contemplation of life, in relation to the larger world context. Daniel’s study was conducted among participants in the Discovery Wilderness Program in 1976 and 2000. The solo phase consisted of two to four days and nights of solitude accompanied by fasting. Two hundred twenty-seven of the 446 participants were asked to recount experiences of their own choosing. Primary data sources included self-administered participant surveys and focus group interviews. Secondary data sources derived from two pilot studies conducted in 1999 and 2000 included written pre- and post-trip questionnaires, taped debriefing sessions, journal entry analysis, reflection papers, and instructors’ observations and field notes. On the one hand, the solo experience was described by the participants as the most significant trip component, providing an opportunity for reflection, introspection, and contemplation in solitude and silence. On the other hand, time alone in silence was perceived by the participants as “dead” or “unproductive,” which led many of them to feel uncomfortable and anxious. As such, solo time entailed mental, physical, emotional, and spiritual challenges that in retrospect were viewed as productive of empowerment and personal growth.

The results of this study suggest that the wilderness solo incorporates the characteristics of significant life events specifically attributed to five factors: (a) A new perspective was gained by the participants through examination of the self in relation to the environment, to others, and to God. (b) It was a new and/or extraordinary experience for most participants when they went on the expedition, and it was unique compared to other life experiences. (c) It took place in beautiful, natural, and inspirational settings. (d) The solo offered mental, physical, emotional, and spiritual challenges. (e) It allowed the opportunity for reflection, introspection, and contemplation in solitude and silence. These studies highlight the significance of the solo experience, specifically linked to beneficial self–reflection; however, the ability to practice self-reflection or the specific personal characteristics linked to engaging positively in these situations was not examined. Therefore Bochniak’s (2007) study is important.

Bochniak focused on individual differences, specifically introversion versus extraversion, as influencing the individual’s perception of the wilderness solo experience. The participants were sixty-four college students, females and males, who participated in a twelve-day backcountry canoe and backpacking trip conducted as part of a wilderness pre-orientation program. Participants filled out a questionnaire measuring extraversion and self-actualization, in relation to wilderness solitude attunement. Participants experienced solitude in four major areas in the following order of frequency: intellectual and spiritual elements; isolation; physical and personal freedom elements; and emotional restoration. The relative novelty of the wilderness solo experience for extraverts was the strongest explanation for their higher wilderness solitude attainment scores compared to introverts, who tended to be more comfortable with being alone. Bochniak concluded by emphasizing the characteristics of the natural environment as greatly contributing to the solo experience. These included tranquility and peacefulness as a result of a lack of intrusions or distractions, opportunities for emotional restoration, and freedom from social constraints that allows individuals to exercise their own free will in thought and action, contributing to the development of individualism and a deep connection with something outside of the self. In addition, silence, described as a lack of interruptions, was considered to be an important part of the solo experience, as was the role of the instructor, although both were not assessed.

There appears to be some consensus that wilderness solos typically have positive outcomes for participants when they have free choice about when and where they spend their period of solitude. For many young people, specifically those who undertake the experience as mandatory in a structured program, the wilderness solo may not have the same positive outcomes. Maxted (2005), who examined 48-hour solo experiences of adolescents over four years, warns about the danger of romanticizing solos as spiritual growth opportunities that for some adolescents are perceived and experienced negatively. Maxted (2005) found a number of fears related to the solo experience and categorized them into fears regarding the wilderness aspects and unexpected encounters with other people, as well as fears of being alone and of the unknown within. Smith (2005b), who has facilitated hundreds of wilderness solos, attributes these fears to the fact that many people do not know how to be alone, finding solitude frightening, boring, or unproductive, emphasizing the importance of preparation and leadership. Kalisch et al. (2011) stressed the difference between being lonely and being alone, with the latter being conceived as essential for mental health and effective leadership.

Whether solitude is experienced as empowering or as a state of loneliness has been linked to the individual’s mindset and coping capabilities. This was the focus of Larson’s (1997) study, differentiating between loneliness as a form of “unhealthy solitude” and being alone as “healthy solitude” involving the ability to cope positively with being alone. Larson assessed these differences by sampling reports from 483 European-American fifth- through ninth-graders. Larson examined the participants’ experience of their companionship and subjective states at random times during the week. He concluded that healthy solitude seems to involve aloneness, not as an end in itself but rather as a temporary withdrawal that complements healthy adjustment in the other social domains of adolescents’ daily lives. This relational perspective is supported by Hollenhorst et al. (1994, p. 234), who developed a scale to measure the psychological dimensions of wilderness solitude that was administered by a survey sent to 298 forest hikers. Based on analysis of participants rating the importance of the different dimensions, the researchers described solitude as a multidimensional concept. In contrast to common notions reflecting solitude in relation to the absence of community, solitude was described as a state of being and, in relation to self, a state of mind. The researchers concluded that the success of wilderness solitude is essentially about “the capacity to cope positively with time spent alone.”

Physical and mental preparation are necessary so that the individual is equipped with the personal resources and strengths for being alone and coping with self and environment in a way that serves the participant’s growth (Knapp and Smith, 2005). Kalisch et al. (2011) suggest the following constructs as influencing the outcome of the wilderness solo: the participant’s receptivity attained by voluntary choice to attend the program; the participant’s expectations; and the instructor, who turned out to greatly influence the participants’ solo experience. Therefore professional facilitation, safety measures, voluntary participation, and proper preparation, during and after the experience, may alter the participant’s perception of the solo.

Maxted (2005, p. 135) also denotes the significance of preparation in order to help participants truly engage with their surroundings, especially for adolescents, as they “need to be jolted out of potential solo boredom in order to connect with nature.” Drawing on reports indicating that adolescents compared to pre-adolescents may receive more benefit from the solo due to better reasoning skills, which allow for deeper self-examination (Larson, 1997), Maxted (2005) concluded that the solo bears the potential to lead to deep thinking if there is appropriate reflective skill-building as a preparation for the wilderness solo. These understandings are supported by Kalisch et al. (2011) in their study that compared experiences of soloists of different ages, finding that younger participants struggled with boredom more than older ones. Therefore Maxted (2005) suggests facilitating “mini-solos,” which take place prior to the “real” wilderness solo and include observational and reflective tasks in the wilderness as optimal preparation. By gradually experiencing solitude in this way, a sense of self that can survive in the absence of immediate social reinforcement is developed and the ability to profit from solitude is enhanced.

### Findings From the Literature

This review sheds light on the solo experience in the wilderness as involving a unique interplay between three basic components that are linked in the reviewed studies to profound personal outcomes, as shown in Table 1.

**TABLE 1:** Summary of empirical studies on the wilderness solo.

Studies	Method and Participants	Silence as experienced in the wilderness solo	Solitude as experienced in the wilderness solo	The Wilderness	The psychological effect
Hammitt and Brown (1984)	Developed and administered a wilderness privacy scale among 109 university students in their quantitative study	Emotional release, and resting the mind from anxiety and mental fatigue	Personal autonomy and self-evaluation	The natural environment devoid of manipulation and domination from others allowed freedom to process information	Cognitive freedom: allowing for the integration of ones thoughts and experiences, which is key for releasing stress and efficient functioning
Hammitt and Madden (1989)	Quantitative study, factor analysis of wilderness privacy scale among 184 overnight backpackers	Lack of human generated noise and intrusion that inhibit peace of mind	Free from constraints, rules and observations from society allowed an acceptable and preferred degree of control and choice over the type of information processed	The natural environment free of man -made noise and intrusions provided a sense of tranquility and peacefulness Directive of one thoughts to what is fascinating	Individualism
Swatton and Potter (1998)	Qualitative study conducted among four adult North American canoeists	Tranquility, peace, and time necessary for self-reflection	Being alone without a sense of judgment by other humans, allowed participants to observe and explore self with greater freedom of expression	The natural environment, devoi of disturbances or sense of rush by other humans, provided an ideal environment for reflection.	Observation and exploration of self as a way to discover and actualize potentials, capabilities, and talents
Hammitt et al. (2001)	Replica of Hammit and Browns quantitative study. Questionnaires administered to 109 university students who spent time alone backpacking in nature.	Tranquility and peace of mind	Personal and social autonomy	Wilderness devoid of manipulation and domination from others	Mental renewal Independence, individuality, and self-evaluation
Daniel (2005)	Multi method pre Dominant qualitative study retrospective study among 227 students participating in a solo experience consisting of two to four days and nights of solitude accompanied by fasting in the Discovery Wilderness Program	The opportunity for reflection, introspection, and contemplation in solitude and silence	Mental, physical, emotional and spiritual challenges that in retrospect were viewed as productive of empowerment and personal growth	The natural environment evoked deep reflection. The beauty and raw power of creation inspired participants to reflect on a creator and was a source of great spiritual inspiration.	The beneficial effect included: a) a broadened understanding of self and the world; (b) a greater awareness of personal strengths and limitations; (c) an enhanced ability to accomplish or at least to try new and difficult tasks; (d) a greater faith and trust in God; (e) a greater awareness of spiritual dimensions; (f) an awareness of the interconnectedness of life; and (g) an increased
Coburn (2006)	Qualitative study to assess transformation among twelve mid age women hiking alone over 2,000 miles on the Appalachian Trail.	Experiencing a sense of being fully present in the moment	Discovering a new and intimate connection with self and nature	Experiencing a sense of wonder, in the vast, timeless, and ever-changing natural environment as well as a sense of vitality, competence and acceptance.	Developing an authentic identity and a desire to be of service
Bochniak (2007)	Quantitative and qualitative designs among sixty four college students, female and male that participated in a twelve-day backcountry canoe and backpacking trip	Emotional restoration	Physical rest and social detachment led to enhanced introspective and spiritual connections.	Tranquility and peacefulness as a result of a lack of intrusions or distractions and freedom from social constraints	Exercise of free will in thought and action, contributing to the development of individualism and a deep connection with something outside of the self
Nicholls (2008)	Qualitative grounded theory inquiry among eighteen youth at risk, participating in a solo experience	Quiet time associated with low levels of noise and a focused attention	The subjective ‘sense’ of solitude included a) a sense of being alone; b) a positive mind frame; c) a personal time perspective and d) focused attention.	A sense of freedom, awe and wonder inspired by being in the wilderness	Gaining an understanding of themselves and their capacity to understand their unresolved and significant domestic concerns Enhanced sense of worth and peace, personal insight, relaxation, and mental clarity
Foster and Borrie (2011)	Qualitative inquiry by interviews conducted with 32 adult overnight backcountry campers	The natural environment, free from intentional human control, provoked new ways of relating to themselves, other humans, powers greater than themselves, and the wild landscape		Mental calm and - reflective thinking brought on by wilderness	New ways of relating to themselves, other humans and nature that evoked spiritual themes
Kalisch et al. (2011)	Quantitative study conducted among 335 first-year college students who participated in solos of various lengths (twenty-four to sixty hours) as part of eighteen-day wilderness programs	Far from human distractions participants were propelled to look inward and reflect on their lives, their experiences, and their relationships with others and God. The environment also contributed a sense of peace and awe as they considered the intricacy and beauty that surrounded them	The enjoyment of being alone, and an appreciation for the opportunity to rest and to reflect on relationships, lives, and future goals but also boredom and challenging	The participants looked forward to having time to relax and to think for an extended time eliciting feeling of peacefulness.	Personal Growth was linked to: a) receptivity, b) optimum degree of stress from the wilderness experience, and c) change of pace and the opportunity for attunement to one’s self and the immediate environment.
Wood (2010)	Qualitative intuitive inquiry method applied among 12 adults who participated in modern-day wilderness solos	Nature was experienced as guiding and enabling, revealing aspects of self through encounters with nature. Inner processes were perceived as part of nature, and interconnected	The opportunity for deep contemplation through reflecting unconscious material in nature	Experiencing a shift in states of consciousness	Developing a more authentic self Discovering a purpose in Life Promoting a sense of wholeness

These components are: (a) silence, experienced in the wilderness solo, not as a seclusion from stimuli but more as allowing the mind to rest from cognitive processing of information and offering the opportunity to experience a form of contemplation by which significant insights regarding the self and the world are attained; (b) solitude, experienced in the wilderness solo as both a challenge and an asset, providing the opportunity for self-discovery, specifically as interconnected to the wider world by which a sense of belonging and purpose are elicited; and (c) the wilderness—providing the tranquility, peace, cognitive freedom and time necessary for significant self-reflection far from daily demands or human interference. The distinct way these components are experienced in the wilderness solo experience are explored in the following section in relation to various theoretical and empirical perspectives, linking these components to psychological health, well-being, and personal growth.

### Silence, Solitude, and the Wilderness in the Context of Personal Growth

#### Silence

The silence of the forest, the peace of the early morning wind moving the branches of the trees, the solitude and isolation of the house of God: these are good

*because it is in silence, and not in commotion, in solitude and not in crowds*,

that God best likes to reveal himself most intimately to men.

—Thomas Merton

Literature in the field of outdoor education discusses silence as a key aspect of the wilderness solo, but the inner experience and effect of silence in the wilderness solo has not been explored. This review reveals silence, experienced in the context of the wilderness solo, as a unique contemplative state. In contrast to common definitions of silence as the absence of sound or communication (Elson, 2001), silence experienced in the wilderness solo has been described as a way to listen and attune to internal insights and knowledge. In the context of the wilderness solo, devoid of external (social, cultural, and mental) stimuli, this silence becomes the fertile ground for authentic self-reflection, self-discovery, and contemplation on one’s personal story, in relation to the greater interconnected web of life. So although Czech philosopher Kohak (1984, p. 127) stated that “we are convinced that truth is in communication, we fear solitude no less than we fear darkness, and have striven strenuously to banish it from our lives,” silence, as experienced in the wilderness solo, invites us to become aware of, and attune to, the sounds, senses, and emotions in both internal and external reality. Wendell Berry (1990, p. 120) describes this as a state in which “one’s inner voices become audible [and] in consequence, one responds more clearly to other lives.” Sardello (2008) calls the direct experience of silence not the absence of something, or a passive state, but more a condition of active receptivity—“living silence.” Integrating this perspective into formulating a spiritual psychology, Sardello (2008, p. 19) states: “Silence is the bountiful source of our sensing our self, and all creation with newfound clarity and intimacy…It is silence that gives our living body its solitude, its oneness with soul and spirit.”

Silence, experienced in the context of the wilderness solo, allows the mental space and cognitive freedom for deep contemplation, akin to the state of *being*. Within his theory of human motivation, needs, and self-actualization, Maslow (1968) described being as an inner state of stillness, linked to contemplation and enjoyment of the inner life (see Maslow, 1970). According to Maslow, states of being are not passive but are rather dynamic, involving growth within—exploring, experiencing, delighting, and enjoying. All these experienced states, while perceived as attitudes of pure being, essentially lead to becoming (Maslow, 1968). Seen as such, the state of being derived from silence, as in the solo experience, is akin to and contributive to the state of mindfulness, defined by Brown and Ryan (2003, p. 822) as “being attentive to and aware of what is taking place in the present.” Howell et al.’s (2011) empirical research study upholds the suggested link between mindfulness, nature connection, and well-being. Their study sought to understand how silence experienced in nature relates to well-being. To assess the connection between nature experience, mindfulness, and well-being, several measures of well-being and wilderness connectedness were administered among 375 undergraduate students. Mindfulness emerged as a significant correlate of nature connectedness, suggesting that the sensory impact of experiences in nature enhance awareness that may foster mindfulness and well-being.

Goodman (1972) wrote: “There is… the fertile silence of awareness, pasturing the soul… the silence of peaceful accord with other persons or communion with the cosmos.” But where does the individual go today to pasture on awareness and commune with the cosmos in a civilization immersed in noise? This review suggests the wilderness solo may provide an optimal environment for the experience and practice of silence, as a mindful state of being, in a contained framework.

#### Solitude

Without great solitude, no serious work is possible.

—Pablo Picasso

Solitude has been defined as “the state of being alone, separated from other people, whether considered a welcome freedom from disturbance or as an unhappy loneliness” (Encarta World English Dictionary, 1999, p. 306). As stated previously, in the wilderness solo, solitude seems to be experienced very differently, as an experience of *being alone together* (Nicholls, 2008, p. 204) or, as described by Cole and Hall (2010) in their inquiry of wilderness privacy, as *being away* from crowds of people, rather than feeling isolated. The inner experience of solitude in the wilderness, described as such, may be attributed to the natural environment enhancing awareness and connections to the wider world. Kohak (1984, p. 39) refers to “the condition of being alone in the presence of a living, familiar world, being willing to listen to it, to see and to understand it. sharing in its feel and meaning.” General conceptions of solitude as being linked to loneliness, a serious problem for many people, has led much contemporary psychological research to focus on alleviating the negative effects of being alone (e.g., Ernst and Cacioppo, 1999). But psychologists interested in the positive aspects of solitude have examined its potential for human development. Winnicott (1958) stated that the fully mature adult is endowed with the potential to engage in solitude for the purpose of controlling anxiety caused by stress and has the capacity to use time alone to reestablish emotional homeostasis. Fiske (1980) described the freedom from distraction, along with focused attention when alone, as providing a unique opportunity to examine and clarify one’s current life situation. Scientific literature has begun to recognize the benefits of solitude as a relief from social stressors; an opportunity for refection and insight; a chance for personal, spiritual, and creative development (Long and Averill, 2003, p. 582); an opportunity to engage in self-selected activities, relatively free of social encumbrances and expectations (Larson, 1990; Burger, 1998); and an opportunity to gain emotional release, self-appraisal, healing, and emotional renewal (Suedfeld, 1982; Storr, 1988).

Empirical research has demonstrated the link between voluntary solitude and well-being. Comfort in being alone was found to be related to lower depression, fewer physical symptoms, and greater life satisfaction (Larson and Lee, 1996). In Larson’s (1997) study following fifth- to ninth-graders for one week, moderate amounts of time spent alone were significantly linked to fewer parent-reported behavioral problems, higher teacher-rated adjustment, lower depression scores, and even higher grade point averages. In an additional study, higher levels of concentration were found among teenagers during time spent by themselves, followed by an increase in cheerfulness and alertness following two hours of solitude. Among adults, comfort in spending time alone was correlated with less sadness, fewer undesirable physical symptoms, and greater overall life satisfaction (Larson and Lee, 1996).

Solitude, experienced in the wilderness solo, seems to involve an awareness of both internal and external connections, by which an individual mindset may expand to a more interconnected one. Koch (1994) describes this in *The Philosophy of Solitude*, stating that one’s consciousness in solitude is not limited to self-focus or inward direction. Solitude experienced as holding the potential for humans to become aware of and connect to broad aspects of existence is aided by silence. “The less one hears of human noise, the more disengaged from people one is, and so the deeper the solitude” (Koch, 1994, p. 20). Nouwen (1981, p. 29) agrees, referring to silence as the vehicle by which the experience of solitude is manifested: “Silence completes and intensifies solitude…silence is the way to make solitude a reality.” Recent literature in the field of psychology demonstrates the significance of both silence and solitude in the context of personal thriving and well-being. Littman-Ovadia (2019) offers a two-dimensional model for understanding and constructing a balanced adult life: doing–being and relationship–solitude. In this model, thriving is attained by active doing (mastery/accomplishment) and by relationships (collaboration/engagement in positive relationships). These comprise two major elements within the common models of well-being (e.g., self-determination theory and the flourish/PERMA theoretical model). However, to live a balanced life, Littman-Ovadia states that the two socially desirable modes of existence—doing and relationships—must be complemented by ‘being’ and ‘solitude’. Both of which are practiced and experienced through the wilderness solo.

#### The Wilderness

And into the forest I go, to lose my mind and find my soul.

— John Muir

The Wilderness Act of 1964 (U.S. Public Law 88-577) mandated congressionally designated wilderness areas, described as providing “outstanding opportunities for solitude” (Hammitt, 1982, p. 293). Although the Wilderness Act clearly specifies the concept of solitude, it does not describe the psychological benefits of these experiences in the wilderness. This review suggests that the natural environment, distant from society and the distractions and pressures of everyday life, can give rise to a unique process of self-discovery, which is elicited by internal–external attunement (Smith, 2005a). Even though experiences in the wilderness are highly individual, specific features in the natural environment seem to contribute to the positive experience of silence and solitude. The capacity to gain benevolent outcomes (e.g., caring and generous behavior) through nature connection has been discussed as *savoring*, which is conceived as the capacity to intentionally attend to positive experiences to enhance positive feelings (Bryant and Veroff, 2007).

An extensive body of research points to the distinct characteristics of beneficial experiences and outcomes of encounters in the wilderness. One example is Snell and Simmonds’s (2012) study on spiritual experiences in nature. They interviewed twenty volunteer participants and found that, as part of their wilderness experience, the participants noticed a switch in their state of mind from analyzing and thinking to being rather meditative and reflective. In this state, the participants found it easier to resolve their personal issues, which led to profound changes in their beliefs, identity, and emotions (Snell and Simmonds, 2012). According to Snell and Simmonds (2012), this process resembles that outlined in Kaplan and Kaplan’s (1989) Attention Restoration Theory, which provides a detailed and empirically sound explanation as to why and how the wilderness can support silence, through restoration of the capacity for directed attention. Based on the findings of their nine-year study focusing on how participants in wilderness programs experienced nature, the researchers developed the concept of “restorative environments” that promote human wellness. Kaplan and Kaplan (1989) defined the distinct characteristics of the natural environment linked to restoration, including (a) being away—from the stresses and responsibilities of daily life that demand direct attention, (b) fascination—as a unique form of effortless attention elicited toward the natural environment, (c) extent—a setting sufficiently rich and coherent that engages the mind and promotes curiosity, and (d) compatibility—the relative simplicity of living in nature and easy functioning as a coherence between the setting and one’s task.

Based on a ten-year study examining the dynamics, impact, and value of the natural environment, Talbot and Kaplan (1986) found that developing a sense of comfort and attunement with the external environment promoted the development of an internal sense of ease and comfort that may support the practice of silence and solitude more than in artificial environments. Furthermore, studies that have compared the settings clearly point to the significant influence of actually being in nature, rather than being exposed to an urban or even virtual natural environment, on complex socio-emotional processes such as dealing with a life problem (Nicholls, 2008). Mayer et al. (2009) conducted studies to investigate whether exposure to nature actually facilitated individuals’ ability to reflect on a life problem. In these studies, participants took a 15-minute walk in an urban or natural environment or watched a 15-minute video of a natural or urban environment. The findings of these studies show a correlation between being in nature and the participants’ ability to reflect on a life problem. Self-reflection was most effective when participants were in the natural environment rather than the virtual natural environment.

The profound effect of the natural environment on this unique state of contemplation and perception has been attributed to specific characteristics of the natural environment, including the vast and boundless landscapes and the beauty and power of nature’s elements. This is shown in Daniel’s (2005) study examining the wilderness solo as a significant life experience. The findings of this study emphasize self-reflection experienced in wilderness solitude as involving not only the self but contemplation on the deepest questions of human existence. What is distinct about this reflection is that it is characterized by a sense of wonder and joy linked to experiencing the power of life and the unity of all. Daniel attributes this unique reflective experience to three specific characteristics of the wilderness that evoke awe and an encompassing perspective: The *beauty* of the places, the *perspective* afforded by being on mountain peaks, and the *power* exhibited by natural elements, such as raging rivers or thunderstorms. Thus, it is not merely enhanced attention resources that support personal growth but also an expanded perspective on life that arises from the experience in nature. This sense of connection to the vast interconnected world has been shown in many cases to inspire a shift in perception, leading to transformative states (Naor and Mayseless, 2017).

So although Thoreau (1995, p. 128) stated that “solitude is not measured by the miles of space that intervene between a man and his fellows,” the natural environment, far from fellow humans, may support the need for “the time and space to re-connect with others and with the greater creation” (Foster and Borrie, 2011, p. 7).

## Discussion

This paper examines the value of the solo experience in wilderness as a modern-day interactive path to practice a unique form of contemplative silence, by which an awareness and attunement to the larger interconnected web of life is cultivated. The studies and accounts reviewed shed light on the distinct characteristics and perceptions of the wilderness solo, involving a unique interplay between three basic components: silence, solitude, and the wilderness, contributing to personal growth, self-discovery, and, in some cases, personal transformation.

The first component—silence—emerged as a unique form of contemplation, experienced in the wilderness as a state of *being*, by which awareness and connection to both internal and external nature was enhanced, specifically as interconnected. The findings of Schlegel et al.’s (2009) study on true self-concept and meaning in life support these understandings, illuminating authenticity and self-knowledge as unique characteristics of the state of being that are essential to the sense of meaning in life.

In the context of psychotherapy, silence has been examined as a form of communication (Levitt, 2001), a way to provide space for an empathic flow between the therapist and client (Elson, 2001), and as a means to listen and convey empathy (Ladany et al., 2004). This paper presents a new perspective underscoring silence not just as a way of listening but as a way of actively discovering aspects of human existence. And as such, it may be through the practice of silence in the wilderness that significant insight and knowledge are attained regarding not only ourselves but the more subtle connections between ourselves and the larger world.

In this way, silence experienced through solo in the wilderness seems to differ from various contemplative approaches to silence that encourage the individual to turn attention away from, or even prevent, the intrusion of sounds, thoughts, emotions, and speech to achieve an inner silence, stillness, and calm (Valle, 2019). In the wilderness solo, silence is experienced as a way to become actively aware of and attuned to the external environment by which an awareness and connection between internal and external nature are developed. From this perspective, silence may elicit a shift from interactions that center on the self to a genuine dialogue between the realms of self-exploration and existential encounters with others, where relational depth blossoms (Lehmann et al., 2019). And as such, it holds the opportunity for self-reflection and contemplation of our personal story, in relation to the greater interconnected web of life.

Based on the works of Heidegger (1962) and Wittgenstein (1958); Bindeman (1981) conceptualized a phenomenology of silence, as a phenomenon that motivates the individual toward self-exploration and self-knowledge. Self-knowledge is attained through silence, by confronting us with an emptiness, mirroring the one who faces it (Bindeman, 1981). In the wilderness, this contemplative state is not only reflecting something about who we are personally but who we are in relation to the large interconnected web of life.

Although the second component, solitude, has been associated with escape or complete isolation from other people, the literature in wilderness therapy indicates that it is experienced very differently in the wilderness. Observations and studies of wilderness users described solitude experienced in the wilderness as involving an awareness and connection to the environment and as such it is experienced less as separation from society and more as connection to the wider world (Heintzman and Mannell, 2003; Bobilya et al., 2005; Wilson, 2011).

Hence, the wilderness solo is an opportunity to be socially separated while developing awareness and intimate relationships with self and the environment through attentive silence. From this perspective, solo time is not the absence of communication but a way of experiencing our unconditional connections and relationships with the world, answering our universal need to belong (Naor and Mayseless, 2019). Interestingly, “feelings of connection to something greater” are delineated by the World Health Organization (2002) as an existential dimension of health. Research has shown that well-being and happiness are associated with defining our “selves” as part of an interconnected whole (Terhaar, 2009). Recent studies relate the psychological value of experiencing a connection with the wider world, contributing to the sense of union and non-duality, which in many instances propelled a sense of meaning and sacredness toward life that was found to be transformative (Heintzman and Mannell, 2003; Bobilya et al., 2005; Naor and Mayseless, 2017).

Solitude experienced in the wilderness is one way to open us up to the various ways of belonging and relating to the world. Seigel (2007) stresses that recognizing life as interconnected leads to well-being by enticing a fundamental shift in our way of living, based on concern for the larger world rather than for our independent selves. Denham-Vaughan and Edmond (2010) describe this shift as a process involving a growing awareness to subtle aspects of our phenomenal field expanding and diffusing our sense of the self–other boundary that defines us. The potential to gain this transformational shift is reinforced by experiencing solitude and silence in the wilderness, for by relating to the environment, the illusion of our separateness as a self becomes dispelled, and we may listen and attune to the external environment, realizing the interconnected unity of our existence.

Larson et al. (1982) described solitude as similar to an ecological niche and suggested that solitude offers potential opportunities, and dangers, either of which may be realized depending on the characteristics of the particular person attempting to thrive there. In this view, the psychological benefits of experiencing solitude depend on the “ecological niche” in which it is experienced. This review suggests the wilderness solo may provide such a niche, which may be used for benevolent outcomes when individuals are offered the proper guidance and support that would enable them to derive personal meaning and growth from the experience of solitude and silence.

The third component of the wilderness solo is the natural environment. The wilderness, perceived as a restorative environment (Kaplan and Kaplan, 1989), is characterized as far from daily demands and human interference, providing the tranquility, peace, and time necessary for self-reflection and contemplation on meaning and purpose in life. The studies reviewed in this paper show that experiencing silence and solitude in the wilderness may yield many more psychological rewards than mere restoration, including the cognitive and social freedom required for self-reflection and significant connection to internal and external reality. Thus, it is not merely enhanced attention resources that enable personal reflection but also a perspective on life gained by a sense of connection that arises from being silent and attentive to nature. Seen as such, the wilderness solo experience may contribute to a shift in perspective from one focused on the self to a more encompassing perspective based on concern for the larger interconnected world (Seigel, 2007). John Muir found this to be so, stating that the wilderness is particularly conducive to enabling people to see themselves as “a small part of the one great union of creation” (Muir, 1938). In fact, the wilderness is the most common trigger for peak experiences characterized by a deep feeling of connection and unity, which in many instances have been linked to major self and life transformations (McDonald and Schreyer, 1991; Naor and Mayseless, 2017).

The facilitated solo experience in the wilderness is one path by which people in modern society can retreat, develop, and remember the human quality and value of silence within a contained framework. And as such, for many people it might be the only option to gain the insight embedded in silence and solitude toward knowing self and personal growth. Kohak (1984, p. 40) states that through solitude in nature, we can “recall the ageless rhythm of nature and. the moral law which our bodies and spirits yet echo beneath the heavy layer of forgetting.”

### Limitations and Future Prospects

This paper focused on the wilderness solo experience, revealed as a unique path to an experienced sense of solitude and silence, by which a deeper knowing of self and the world is gained. Within the limits of this paper and in light of our objective to gain a better understanding regarding the value of silence and solitude to personal growth, additional components of the experience were not reviewed. Assessing additional components (e.g., experiencing the solo with others; personal history, specifically in connection with experiences in the wilderness; personal characteristics that may support or limit the ability to be alone in silence; therapeutic objectives that may focus more on coping with being alone rather than gaining personal insight; professional conduct, including the alliance and trust that provides sufficient support; and adequate preparation and integration) would contribute to our understanding of the phenomena toward a general conceptualization of the solo experience as a nature - based intervention that promotes health and well-being as provided by Irvine et al. (2020) in their model for nature-based interventions. Further inquiry is required to gain a better understanding specifically regarding the therapeutic value of experiencing silence toward personal, social, and environmental health. We hope that the potential embedded in the solo experience for personal development as reviewed in this paper will provide new prospects for therapeutic interventions that involve periods of silence and solitude.

## Author Contributions

LN and OM conceived of the presented idea. LN reviewed the literature in the field and put the main ideas into writing. OM advised LN on the development of the main ideas and provided critical revision of the manuscript. Both authors discussed the main objective and contribution of the manuscript resulting in the final manuscript. Both authors contributed to the article and approved the submitted version.

## Conflict of Interest

The authors declare that the research was conducted in the absence of any commercial or financial relationships that could be construed as a potential conflict of interest.

## References

[B1] Berry T. (1990). *The Dream of the Earth.* San Francisco: Sierra Club Books.

[B2] Bindeman S. (1981). *Heidegger and Wittgenstein: The Poetics of Silence.* Washington, DC: University Press of America.

[B3] Bobilya A. (2005). “Wilderness, solitude, and monastic traditions,” in *Exploring the Power of Solo, Silence, and Solitude*, eds Knapp C. Smith T. (Boulder, CO: Association for Experiential Education), 61–74.

[B4] Bobilya A. J. McAvoy L. H. Kalisch K. R. (2005). “Lessons from the field: participant perceptions of a multi-day wilderness solo,” in *Exploring the Power of Solo, Silence, and Solitude*, eds Knapp C. Smith T. (Boulder, CO: Association for Experiential Education), 103–121.

[B5] Bochniak J. (2007). *The Relationships Between Extraversion, Self-Actualization, and Wilderness Solitude Attainment for Participants in a Wilderness Solo Experience.* Dissertation, University of New York College, New York, NY.

[B6] Brown K. W. Ryan R. M. (2003). The benefits of being present: mindfulness and its role in psychological well-being. *J. Pers. Soc. Psychol.* 84 822–845. 10.1037/0022-3514.84.4.822 12703651

[B7] Bruneau T. Ishii S. (1988). Communicative silences: east and west. *Word Commun.* 17 1–33.

[B8] Bryant F. B. Veroff J. (2007). *Savoring: A New Model of Positive Experience.* Mahwah, NJ: Lawrence Erlbaum Associates.

[B9] Burger J. M. (1998). “Solitude,” in *Encyclopedia of Mental Health* [Vol. 3], ed. Friedman H. S. (San Diego, CA: Academic Press), 563–569.

[B10] Burns G. W. (2005). “Naturally happy, naturally healthy: the role of the natural environment in well-being,” in *The Science of Well-Being*, eds Huppert F. A. Baylis N. Keverne B. (New York, NY: Oxford University Press), 404–431. 10.1093/acprof:oso/9780198567523.003.0016

[B11] Byrnit J. (2006). Primate theory of mind: a state of the art review. *J. Anthropol. Psychol.* 17 5–48.

[B12] Coburn M. J. (2006). *Walking Home: Women’s Transformative Experiences in the Wilderness of the Appalachian Trail.* dissertation, Institute of Transpersonal Psychology, Palo Alto, CA 10.1037/e538962007-001

[B13] Cole D. N. Hall T. E. (2010). Privacy functions and wilderness recreation: use density and length of stay effects on experience. *Ecopsychology* 2 67–75. 10.1089/eco.2010.0003

[B14] Daniel B. (2005). “The life significance of a wilderness solo experience,” in *Exploring the Power of Solo, Silence, and Solitude*, ed. Knapp C. Smith T. (Boulder, CO: Association for Experiential Education), 85–102. 10.1300/j015v15n03_07

[B15] Denham-Vaughan J. Edmond V. (2010). The value of silence. *Gestalt J. Austral. New Zeal.* 6 5–19.

[B16] Dénommé -Welch S. Rowsell J. (2017). Epistemologies of silence. *Brock Educ. J.* 27 10–25. 10.26522/brocked.v27i1.622

[B17] Elson M. (2001). Silence, its use and abuse: a view from self-psychology. *Clin. Soc. Work J.* 29 351–360. 10.1023/A:1012215213461

[B18] Encarta World English Dictionary (1999). *Microsoft Corporation.* London: Bloomsbury Publishing Plc.

[B19] Ernst J. M. Cacioppo J. T. (1999). Lonely hearts: psychological perspectives on loneliness. *Appl. Prevent. Psychol.* 8 1–22. 10.1016/s0962-1849(99)80008-0

[B20] Fiske M. (1980). “Changing hierarchies of commitment in adulthood,” in *Themes of Work and Love in Adulthood*, eds Smelser N. J. Erikson E. H. (Cambridge, MA: Harvard University Press), 238–264.

[B21] Foster I. M. Borrie W. T. (2011). *A Phenomenology of Spiritual Experiences in Wilderness: Relating Self, Culture and Wilderness*. Available online at: https://www.semanticscholar.org/paper/A-Phe nomenology-of-Spiritual-Experiences-in-Self%2CFoster-Borrie/0ac022cc984c2a0b40809d26962e26c5baeadfd1

[B22] Fredrickson L. Anderson D. (1999). A qualitative exploration of the wilderness experience as a source of spiritual inspiration. *J. Environ. Psychol.* 19 21–39. 10.1006/jevp.1998.0110

[B23] Gennep A. (1960). *The Rites of Passage (Vizedom, M. B., and Caffee, G. L., (Trans.).* Chicago, IL: University of Chicago.

[B24] Gibbens R. (1991). The bliss of solitude. *J. Advent. Educ. Outdoor Leadersh.* 8 21–23.

[B25] Goodman P. (1972). *Speaking and Language: Defense of Poetry.* New York: Random House

[B26] Hammitt W. E. (1982). Cognitive dimensions of wilderness solitude. *Environ. Behav.* 14 478–493. 10.1177/0013916582144005

[B27] Hammitt W. E. Backman K. F. Davis T. J. (2001). Cognitive dimensions of wilderness privacy: an 18-year trend comparison. *Leisure Sci.* 23(4) 285–292. 10.1080/01490400152809124

[B28] Hammitt W. E. Brown G. F. (1984). Functions of privacy in wilderness environments. *Leisure Sci.* 6 151–166. 10.1080/01490408409513028

[B29] Hammitt W. E. Madden M. A. (1989). Cognitive dimensions of wilderness privacy: a field test and further explanation. *Leisure Sci.* 11 293–301 10.1080/01490408909512228

[B30] Heidegger M. (1962). *Being and Time (J. Macquarrie and E. Robinson, Trans.).* New York, NY: Harper and Row.

[B31] Heintzman P. Mannell R. C. (2003). Spiritual functions of leisure and spiritual well-being: coping with time pressure. *Leisure Sci.* 25 207–230. 10.1080/01490400306563

[B32] Hollenhorst S. Frank E. Watson A. (1994). “The capacity to be alone: wilderness solitude and growth of the self,” in *International Wilderness Allocation, Management, and Research*, eds Hendee J. C. Martin V. G. (Fort Collins, CO: International Wilderness Leadership Foundation), 234–239.

[B33] Howell A. J. Dopko R. L. Passmore H. A. Buro K. (2011). Nature connectedness: associations with well-being and mindfulness. *Pers. Individ. Diff.* 51 166–171. 10.1016/j.paid.2011.03.037

[B34] Irvine K. N. Marselle M. R. Melrose A. Warber S. L. (2020). Group outdoor health walks using activity trackers: measurement and implementation insight from a mixed methods feasibility study. *Int. J. Environ. Res. Public Health* 17(7), 2515. 10.3390/ijerph17072515 32272603 PMC7177624

[B35] Jung C. G. (1951). “Fundamental questions of psychotherapy,” in *The Collected Works of C. G. Jung*, ed. Read H. (Princeton, NJ: Princeton University Press), 111–125. 10.1515/9781400851003.111

[B36] Kalisch K. R. Bobilya A. J. Daniel B. (2011). The outward bound solo: a study of participants’ perceptions. *J. Exp. Educ.* 34 1–18. 10.1177/105382591103400102

[B37] Kaplan R. Kaplan S. (1989). *The Experience of Nature: A Psychological Perspective.* New York, NY: Cambridge University Press.

[B38] Kemerer D. (2016). How to use intentional silence. *Nurs. Stand.* 31 42–44. 10.7748/ns.2016.e10538 27794696

[B39] Knapp C Smith T. (eds) (2005). *Exploring the Power of Solo, Silence, and Solitude.* Boulder, CO: Association for Experiential Education.

[B40] Koch P. (1994). *Solitude: A Philosophical Encounter.* Chicago, IL: Open Court Press.

[B41] Kohak E. (1984). *The Embers and Stars: A Philosophical Inquiry into the Moral Sense of Nature.* Chicago, IL: The University of Chicago Press.

[B42] Ladany N. Hill C. Thompson B. O’Brien K. (2004). Therapist perspectives on using silence in therapy: a qualitative study. *Counsel. Psychother. Res.* 4 80–89. 10.1080/14733140412331384088

[B43] Larson R. Csikszentmihalyi M. Graef R. (1982). “Time alone in daily experience: loneliness or renewal,” in *Loneliness: A Sourcebook of Current Theory, Research, and Therapy*, eds Peplau L. A. Perlman D. (New York, NY: Wiley-Interscience), 40–53.

[B44] Larson R. Lee M. (1996). The capacity to be alone as a stress buffer. *J. Soc. Psychol.* 136 5–16. 10.1080/00224545.1996.9923024 8851444

[B45] Larson R. W. (1990). The solitary side of life: an examination of the time people spend alone from childhood to old age. *Dev. Review* 10 155–183. 10.1016/0273-2297(90)90008-r

[B46] Larson R. W. (1997). The emergence of solitude as a constructive domain of experience in early adolescence. *Child Dev.* 68 80–93. 10.2307/1131927 9084127

[B47] Lehmann O. V. (2016). Something blossoms in between: silence-phenomena as bordering notions in psychology. *Integr. Psychol. Behav. Sci.* 50 1–13. 10.1007/s12124-015-9321-7 26232279

[B48] Lehmann O. V. Kardum G. Klempe H. (2019). The search for inner silence as a source for eudemonia. *Br. J. Guid. Counsel.* 47 180–189. 10.1080/03069885.2018.1553295

[B49] Levitt H. (2001). Sounds of silence in psychotherapy: the categorization of clients’ pauses. *Psychother. Res.* 11 295–309. 10.1080/713663985

[B50] Littman-Ovadia H. (2019). Doing–being and relationship–solitude: a proposed model for a balanced life. *J. Happiness Stud.* 20 1953–1971. 10.1007/s10902-018-0018-8

[B51] Long C. Averill J. (2003). Solitude: an exploration of the benefits of being alone. *J. Theory Soc. Behav.* 33(1), 21–44 10.1111/1468-5914.00204

[B53] Marcandonatou O. (1998). “The experience of being silent,” in *Phenomenological Inquiry in Psychology: Existential and Transpersonal Dimensions*, ed. Valle R. (New York, NY: Plenum Press), 309–320. 10.1007/978-1-4899-0125-5_14

[B54] Maslow A. H. (1968). *Toward a Psychology of Being.* New York, NY: Wiley.

[B55] Maslow A. H. (1970). *Motivation and Personality*, 2nd ed New York, NY: Harper & Row.

[B56] Maxted J. (2005). “Coming home: adolescents and the nature-based solo,” in *Exploring the Power of Solo, Silence, and Solitude*, eds Knapp C. Smith T. (Boulder, CO: Association for Experiential Education), 61–74.

[B57] Mayer F. S. Frantz C. M. Bruehlman-Senecal E. Dolliver K. (2009). Why is nature beneficial? The role of connectedness to nature. *Environ. Behav.* 41 607–643. 10.1177/0013916508319745

[B58] McDonald B. Schreyer R. (1991). “Spiritual benefits of leisure: participation and leisure settings,” in *Benefits of Leisure*, eds Driver B. L. Brown P. J. Peterson G. L. (Venture, PA: State College), 179–194.

[B59] Muir J. (1938). *John of the Mountains: the Unpublished Journals of John Muir.* Boston, MA: Houghton Mifflin press.

[B60] Naor L. Mayseless O. (2017). How personal transformation occurs following a single peak experience in nature: a phenomenological account. *J. Hum. Psychol.* 57 13–24. 10.1177/0022167817714692

[B61] Naor L. Mayseless O. (2019). The therapeutic value of experiencing spirituality in nature. *Spirit. Clin. Pract.* 7 114–133. 10.1037/scp0000204

[B62] Nicholls V. (2008). Busy doing nothing: researching the phenomenon of ‘quiet time’ in a challenged-based therapy program. *J. Exp. Educ.* 26 8–24.

[B63] Norris J. (2011). Crossing the threshold mindfully: exploring rites of passage models in adventure therapy. *J. Advent. Educ. Outdoor Learn.* 11 109–126. 10.1080/14729679.2011.633380

[B64] Nouwen H. J. M. (1981). *The Way of the Heart.* New York. NY: Ballantine Press.

[B65] Picard M. (2002). *The World of Silence.* Wichita, KS: Eighth Day Books.

[B66] Sabini M. (2008). “C. G. Jung, the earth has a soul,” in *C. G. Jung on Nature, Technology and Modern Life*, ed. Sabini M. (Berkeley, CA: North Atlantic Books), 67–89.

[B67] Sardello R. (2008). *Silence: The Mystery of Wholeness.* Berkeley, CA: Goldenstone Press.

[B68] Schlegel R. J. Hicks J. A. Arndt J. King L. (2009). Thine own self: true self-concept accessibility and meaning in life. *J. Pers. Soc. Psychol.* 96 473–490. 10.1037/a0014060 19159144 PMC4714566

[B69] Seigel D. J. (2007). *The Mindful Brain.* New York: W. W. Norton Press.

[B70] Shanahan D. F. Astell-Burt T. Barber E. A. Brymer E. Cox D. T. C. Dean J. (2019). Nature–based interventions for improving health and wellbeing: the purpose, the people and the outcomes. *Sports* 7:141. 10.3390/sports7060141 31185675 PMC6628071

[B71] Smith T. (2005a). “Going outside to go inside: frameworks for the solo experience,” in *Exploring the Power of Solo, Silence, and Solitude*, eds Knapp C. Smith T. (Boulder, CO: Association for Experiential Education), 3–18.

[B72] Smith T. (2005b). “Solitude for growth and healing: 10 memories,” in *Exploring the Power of Solo, Silence, and Solitude*, eds Knapp C. Smith T. (Boulder, CO: Association for Experiential Education), 273–280.

[B73] Snell T. L. Simmonds J. G. (2012). “Being in that environment can be very therapeutic”: spiritual experiences in nature. *Ecopsychology* 4 326–335. 10.1089/eco.2012.0078

[B74] Storr A. (1988). *Solitude: A Return to the Self.* New York, NY: Free Press.

[B75] Suedfeld P. (1982). “Aloneness as a healing experience,” in *Loneliness: A Sourcebook of Current Theory, Research, and Therapy*, eds Peplau L. A. Perlman D. (New York, NY: Wiley-Inter science), 13–46.

[B76] Swatton A. Potter T. (1998). The personal growth of outstanding canoeists resulting from extended solo canoe expeditions. *Pathways* 9 13–16.

[B77] Talbot J. Kaplan S. (1986). Perspectives on wilderness: re-examining the values of extended wilderness experiences. *J. Environ. Psychol.* 6 177–188. 10.1016/s0272-4944(86)80021-4

[B78] Terhaar T. L. (2009). Evolutionary advantages of intense spiritual experience in nature. *J. Study Religion Nat. Cult.* 3 303–339. 10.1558/jsrnc.v3i3.303

[B79] Thoreau H. (1995). *Walden; Or, Life in the Woods (Unabridged replication, 1854, Ticknor & Fields, Boston ed.).* New York, NY: Dover Publications Inc.

[B80] Valle R. (2019). Toward a psychology of silence. *Hum. Psychol.* 47 219–261. 10.1037/hum0000120

[B81] Vaughan C. Klimo J. (2016). “Intuition in the room: a phenomenological study of psychotherapists’ intuitive experience,” in *The Changing Faces of Therapy: Evolving Perspectives in Clinical Practice and Assessment*, ed. Valle R. (Alameda, CA: Argosy University), 117–154.

[B82] Wilson M. (2011). Encounters with nature as a path of self- realization: a meaning making framework. *J. Transpers. Res.* 3, 11–29

[B83] Winnicott D. W. (1958). The capacity to be alone. *Int. J. Psychoanal.* 39:416. 13610513

[B84] Wittgenstein L. (1958). *Philosophical Investigations.* Oxford: Basil Blackwell.

[B85] Wood R. (2010). *Psycho-Spiritual Transformation Experienced by Participants of 17 Modern Wilderness Rites of Passage Uests: An Intuitive Inquiry.* Dissertation, Institute of Transpersonal Psychology, Palo Alto, CA.

[B86] World Health Organization (2002). Available online at: http://www.who.int/iris/handle/10665/77777 (Accessed August 1, 2020).

